# A glucagon analogue decreases body weight in mice via signalling in the liver

**DOI:** 10.1038/s41598-021-01912-0

**Published:** 2021-11-19

**Authors:** Charlotte E. Hinds, Bryn M. Owen, David C. D. Hope, Philip Pickford, Ben Jones, Tricia M. Tan, James S. Minnion, Stephen R. Bloom

**Affiliations:** Section of Investigative Medicine, Department of Metabolism, Digestion, and Reproduction, Imperial College London, London, W12 0NN UK

**Keywords:** Fat metabolism, Pharmacology

## Abstract

Glucagon receptor agonists show promise as components of next generation metabolic syndrome pharmacotherapies. However, the biology of glucagon action is complex, controversial, and likely context dependent. As such, a better understanding of chronic glucagon receptor (GCGR) agonism is essential to identify and mitigate potential clinical side-effects. Herein we present a novel, long-acting glucagon analogue (GCG104) with high receptor-specificity and potent in vivo action. It has allowed us to make two important observations about the biology of sustained GCGR agonism. First, it causes weight loss in mice by direct receptor signalling at the level of the liver. Second, subtle changes in GCG104-sensitivity, possibly due to interindividual variation, may be sufficient to alter its effects on metabolic parameters. Together, these findings confirm the liver as a principal target for glucagon-mediated weight loss and provide new insights into the biology of glucagon analogues.

## Introduction

Glucagon is a 29-amino acid pancreatic hormone that maintains euglycemia during fasting through action on the liver. It drives gluconeogenesis and glycogenolysis and, in this capacity, can act as a counter-regulatory hormone to insulin^1^. However, the biology of glucagon is more complex than that of a simple nutritionally regulated glucoregulatory hormone. For example, it is also secreted in response to a range of physiological stressors^2^. In addition, chronic exposure to high levels of glucagon, as originally described in patients with glucagonomas, can induce anorexia and potentially muscle mass loss^3,4^. Indeed, glucagon was shown to mediate weight loss in humans in 1957^5^. Subsequent studies demonstrated that this effect may be due to glucagon’s capacity to increase energy expenditure^6–8^. As such, it has been suggested that in the correct context, chronic glucagon receptor (GCGR) signalling may actually be of benefit to obese and diabetic patients despite its hyperglycaemic effect^9^. Indeed, several bioactive peptides which include GCGR agonism as a deliberate component of their pharmacodynamic profile are being developed which show promise for the treatment of metabolic syndromes in humans^10–12^. In many cases these act as functional analogues of oxyntomodulin (OXM), which also stimulates the glucagon-like peptide-1 receptor (GLP-1R) to maximise weight loss and augment insulin secretion^13^. However, optimizing the clinical utility of GCGR/GLP-1R dual-therapy is hampered by our incomplete understanding of the biology of these hormones.

The GCGR is expressed predominantly in hepatocytes and to a lesser extent within the kidneys, adipose tissues, enteroendocrine system, and select regions of the brain^14,15^. Some of the biological effects of glucagon, however, may be indirectly mediated by secondary effectors, such as activation of the sympathetic nervous system, upregulation of other hormones like hepatokines, or through liver-mediated changes in specific metabolites^16,17^. Robust regulatory systems further complicate the interpretation of specific glucagon action, especially in chronic pharmacological settings or where the metabolic status of the organism may influence glucagon-sensitivity.

Here, we report on the actions of a novel glucagon analogue (GCG104) with GCGR-specificity and a lipidated tail. Using tissue-specific knockout mice, we show that it mediates weight loss in mice by acting on the GCGR in the liver. However, our experiments have also revealed that subtle differences in GCG104 dose/sensitivity can alter its biology. Our findings highlight the importance of considering agonist dose and sensitivity, both in mechanistic studies and potential clinical applications of glucagon receptor agonists.

## Materials and methods

### Animals

All animal procedures were approved by the UK Animals (Scientific Procedures) Act 1986 and carried out in compliance with the Animal Research: Reporting of Vivo Experiments (ARRIVE) guidelines. Experiments were also approved by the Animal Welfare Ethical Review Board (AWERB) of Imperial College, London and all methods were conducted in accordance with the relevant guidelines and regulations stipulated by Imperial College, London AWERB.

All mice were single-housed under standard conditions in a 12-h light/dark cycle and fed standard chow diet (RM1(E), Special Diets Services, UK) with water available ad libitum*.* Studies commenced from 10 weeks of age in male mice. For determination of GCG104 pharmacokinetics, 8-week-old mixed-sex Wistar rats (Charles River, UK) were used rather than mice because the larger circulating blood volume allowed serial-sampling and thus a reduction in animal usage.

To generate animals with whole-body deletion of the *Gcgr, C57BL/6J Gcgr*^*flox/flox*^ mice^18^ were first crossed with hemizygous *Actβ*^*-Cre-ERT2*^ mice. Then, at 4 weeks, both *Actβ*^*-(Cre-ERT2)*+^:*Gcgr*^*flox/flox*^ and *Actβ*^*-(Cre-ERT2)-*^:*Gcgr*^*flox/flox*^ animals were orally gavaged with 100 mg/kg tamoxifen in corn oil (Thermo Fisher, USA) for 5 consecutive days, generating *Gcgr*^*-/-*^ mice (herein defined as ‘cKO’ mice) and control mice, respectively. To generate animals with deletion of the *Gcgr* specifically in hepatocytes, *Gcgr*^*flox/flox*^ mice were crossed with hemizygous *Albumin-Cre* mice (Jackson Labs Stock #003574) to generate *Gcgr*^*flox/flox*^*:Alb-Cre*^+^ mice (herein defined as ‘hKO’ mice) and control *Gcgr*^*flox/flox*^*:Alb-Cre*^*-*^ mice. Confirmation of both knockouts was determined by quantitative PCR (Supplementary Fig. 1) as detailed below.

### Quantitative PCR (qPCR)

Quantification of *Gcgr* expression in liver was achieved by standard methods. Briefly, cDNA was synthesized from liver RNA using a High-Capacity cDNA Reverse Transcription Kit (Thermo Fisher, USA). SYBR Green (Bio-Rad) was used to amplify the *Gcgr* gene using validated gene-specific primers (Sigma Aldrich). Data are presented as relative expression using *Cyclophilin* as an internal control.

### GCG104 administration

GCG104 was synthesised by Wuxi AppTec (Wuhan, China) and was 88% pure. For all mouse studies, animals received GCG104 or saline at 9.00am every day for 11 consecutive days. Randomisation of treatment groups was stratified by body weight. Food and body weight measurements were taken at the same time as injection.

### Determination of pharmacokinetics

Rats were administered 0.5 mg of GCG104 prepared in water for injection via 100 µl subcutaneous injection. 100 µl blood samples were collected via superficial tail venesection using EDTA coated microvettes (16.444; Sarstedt, USA) prior to injection and at 3, 24, 48, 72 and 168 h after injection. Blood samples were immediately frozen on ice and then spun at 8000 × g for 10 min at 4 °C. The plasma was then collected and stored at − 20 °C when not in use.

Glucagon N-terminal-like immunoreactivity was measured using an in-house radioimmunoassay to determine peptide concentrations in the rat plasma. The assay was performed in a total volume of 700 µl of 0.06 M phosphate EDTA buffer containing 0.3% BSA. Antiserum (RA553/3) was produced in rabbits against the purified glucagon(1–16) fragment which specifically cross-reacted with the N-terminal glucagon sequence found in GCG104. I125 glucagon was produced by direct iodination and purified by reverse phase high pressure liquid chromatography. All samples were assayed in duplicate and the standard curved was set using 5 pmol/ml GCG104 prepared in assay buffer. Incubation occurred over 3 nights at 4 °C and then the samples were separated into free and antibody-bound label using charcoal. The pellet and supernatant were counted for 180 s in a γ-counter and the ratio of free to bound label calculated. The plasma peptide concentration was calculated using two phase exponential decay with respect to the relevant standard curve (Prism, V9.0, GraphPad Software, USA).

### Glucose tolerance tests

Mice were fasted for 4.5 h with ad libitum access to water. Baseline blood samples were taken from the tail vein. Glucose was administered at 2 g/kg via an intraperitoneal injection and the second blood glucose measurements were taken 15 min later.

### Body composition analysis via magnetic resonance imaging (MRI)

Body composition was measured by EchoMRI (EchoMRI LLC, Texas).

### Cell culture and cyclic AMP accumulation assays

HEK293T cells were transiently transfected with plasmids encoding mGLP-1R (MG57391-UT; Sino Biological, USA) or mGCGR (MG57952-UT; Sino Biological, USA) for 24 h before the assay. 0.1 µg receptor plasma DNA + 0.9 µg pcDNA3.1 carrier DNA were transfected per 12-well plate using Lipofectamine 2000, according to the manufacturer’s instructions. Cells were detached and treated with the indicated agonist concentration in the presence of 100 µM IBMX for 30 min at 37 °C. cAMP was determined by HTRF using Cisbio cAMP Dynamic 2 kit. Responses were analysed by 3-parameter logistic fitting, with E_max_ constraints applied when appropriate (i.e. for incomplete mGLP-1R responses).

### Statistical analysis

Statistical analyses were performed using GraphPad Prism V9.0 (GraphPad Software, USA). All data is presented as mean ± SEM. For multiple group comparison analysis, two-way ANOVA was used, followed by Sidak post hoc testing. Repeated-measures ANOVA were used where appropriate, followed by Tukey post hoc testing. Where two independent groups were compared, an unpaired student’s t-test was used. A *p* value < 0.05 was considered significant.

## Results and discussion

### GCG104 is a selective and potent glucagon analogue

**Figure 1:**
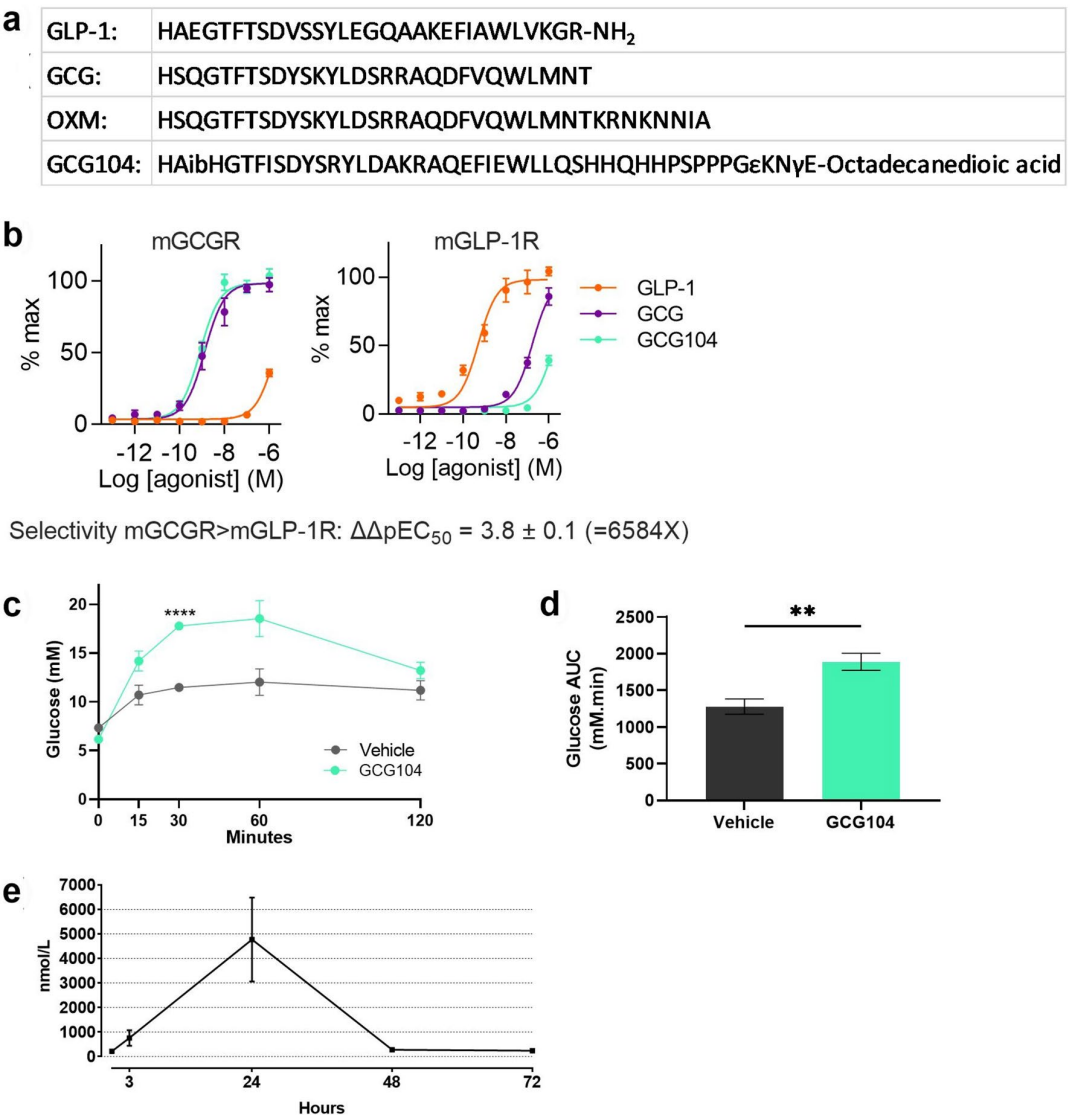
Pharmacological profile of GCG104 in vitro and in vivo. (**a**) Primary structure of native GLP-1, glucagon (GCG), oxyntomodulin (OXM) and GCG104. Sequences are given in standard single amino acid code with Aib denoting alpha-amino isobutyric acid. (**b**) % max cAMP accumulation following GCG104, GLP-1 or glucagon treatment to HEK293 cells transiently expressing mGCGR or mGLP-1R (n = 4 for all tests). (**c**) Glucose response following vehicle or 10 nmol/kg GCG104 injection in 2 h fasted wild-type mice (n = 4 per group; 2-way ANOVA with Sidak post hoc test). (**d**) Corresponding glucose area under the curve for graph c (unpaired *t* test). (**e**) Pharmacokinetic profile of GCG104 in rats following 0.5 mg subcutaneous injection (n = 5 per group). All data presented as mean ± SEM. *p* values indicated by **p < 0.01, ****p < 0.0001.

Native glucagon has a half-life of approximately 2 min in rodents^19^. To study the effects of sustained GCGR activation, we developed a glucagon analogue (GCG104) through modification of OXM with the aim of reducing its GLP-1R activity and decreasing its rate of clearance. Select amino acid substitutions were made and a C18 lipidated tail was added (Fig. 1a). In vitro assessment of this molecule demonstrated that its potency at the mouse GCGR (mGCGR) is similar to that of native glucagon (Fig. 1b). However, GCG104 showed even lower mGLP-1R potency than native glucagon, with EC_50_ comparisons showing over 6000-fold selectivity for mGCGR over mGLP-1R compared to the respective endogenous ligands (Fig. 1b). In vivo, GCG104 plasma levels remained elevated at 24 h, when native glucagon would have cleared, and increased blood glucose levels following intraperitoneal injection (Fig. 1c–e). Together, these data demonstrate that GCG104 is highly specific to the mGCGR, long lasting, and has potent glucagon-like activity in vivo.

### GCG104 reduces body weight in mice via the glucagon receptor

**Figure 2:**
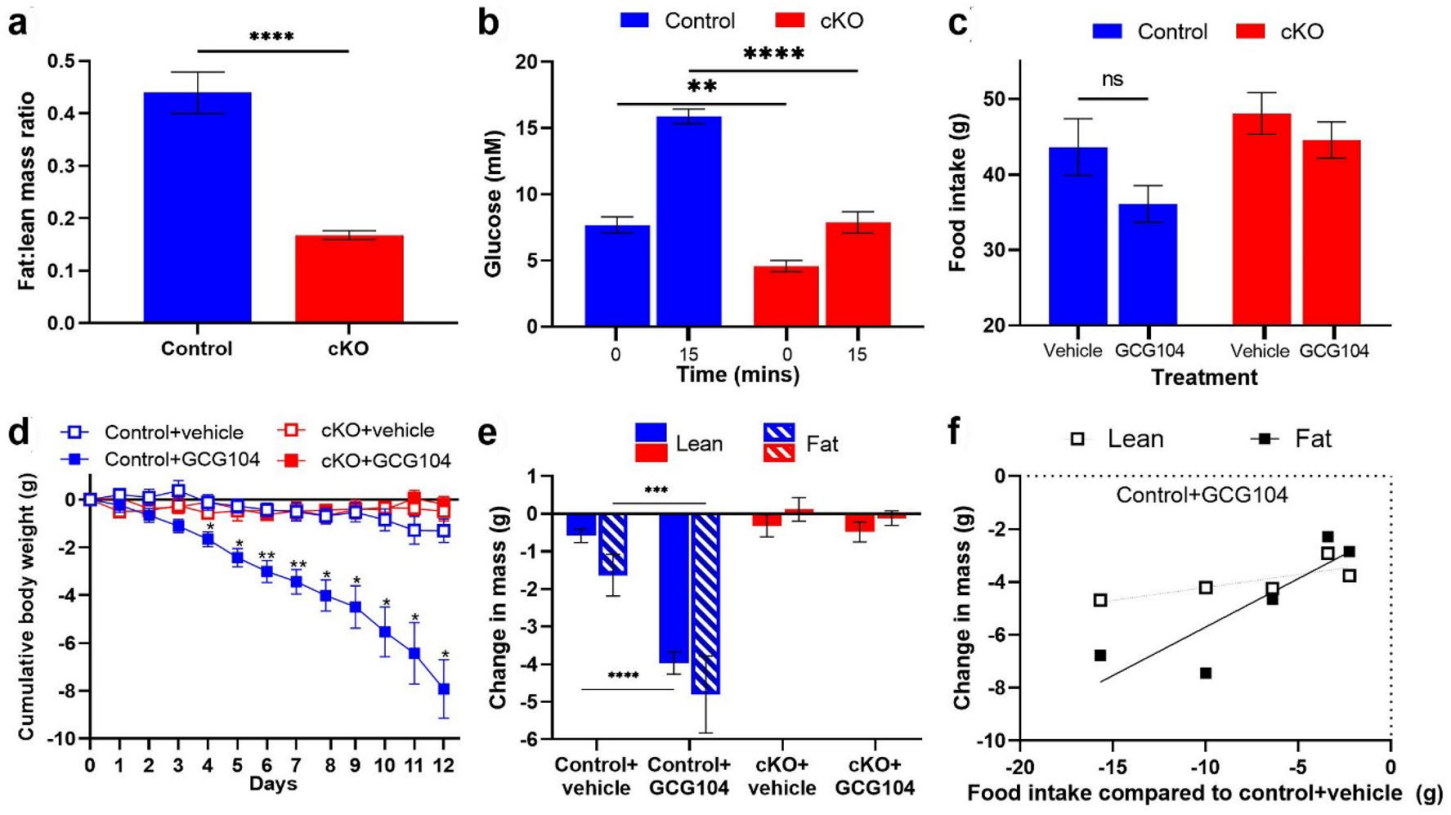
GCG104 reduces body weight in mice via the GCGR. (**a**) Fat:lean mass ratio of the cKO animals (n = 10) and controls (n = 11; unpaired *t* test). (**b**) Baseline and 15-min glucose response readings for an IPGTT (2 g/kg) in the cKO (n = 5) and control animals (n = 6; two-way ANOVA with Sidak post hoc test). (**c**) 12-day cumulative food intake for control and cKO animals injected daily with vehicle or 7.5 nmol/kg GCG104 (n = 5 per group; two-way ANOVA with Sidak post hoc test). (**d**) 12-day cumulative body weight change in control and cKO animals injected daily with vehicle or 7.5 nmol/kg GCG104. (n = 5 per group; repeated measures 2-way ANOVA with Tukey’s post hoc test). (**e**) 12-day change in body composition for control and cKO animals dosed with vehicle or 7.5 nmol/kg GCG104 (n = 5 per group; two-way ANOVA with Sidak post hoc test). (**f**) Correlation between change in body composition and reduction in food intake compared to vehicle-dosed control mice in GCG104-dosed control mice (Pearson’s *r*: fat = 0.87; lean = 0.79). *p* values indicated by ns p > 0.05, *p < 0.05, **p < 0.01, ***p < 0.001 ****p < 0.0001. All data presented as mean ± SEM.

In order to fully validate the GCGR-dependent actions of GCG104, we generated animals with conditional whole-body deletion of the *Gcgr* (‘cKO’). EchoMRI demonstrated that these animals had a reduced fat:lean mass ratio (Fig. 2a). They also showed reduced fasting and stimulated blood glucose during an intraperitoneal glucose tolerance test (Fig. 2b). These findings are consistent with previous reports of whole-body *Gcgr* knockout mice^20,21^. Next, we established a dose of GCG104 (7.5 nmol/kg) that reduced food intake in some but not all animals; a proposed ‘peri-anorectic’ dose (Fig. 2c). Using this dose, we aimed to determine the effects of chronic GCGR agonism on body weight and body composition.

Daily administration of GCG104 significantly reduced body weight in control but not cKO animals (Fig. 2d and Supplementary Fig. 2a), thus demonstrating GCGR-specific weight lowering action in vivo. EchoMRI analysis demonstrated that weight loss was a result of a reduction in both fat mass and lean mass (Fig. 2e and Supplementary Fig. 3a), as has been reported for other glucagon analogues^16,22^. Interestingly, correlations between food intake and changes in body composition (Fig. 2f) suggest that the reduction of fat mass may be driven by reduced energy intake.

### GCG104 reduces lean mass via hepatocyte signalling

**Figure 3:**
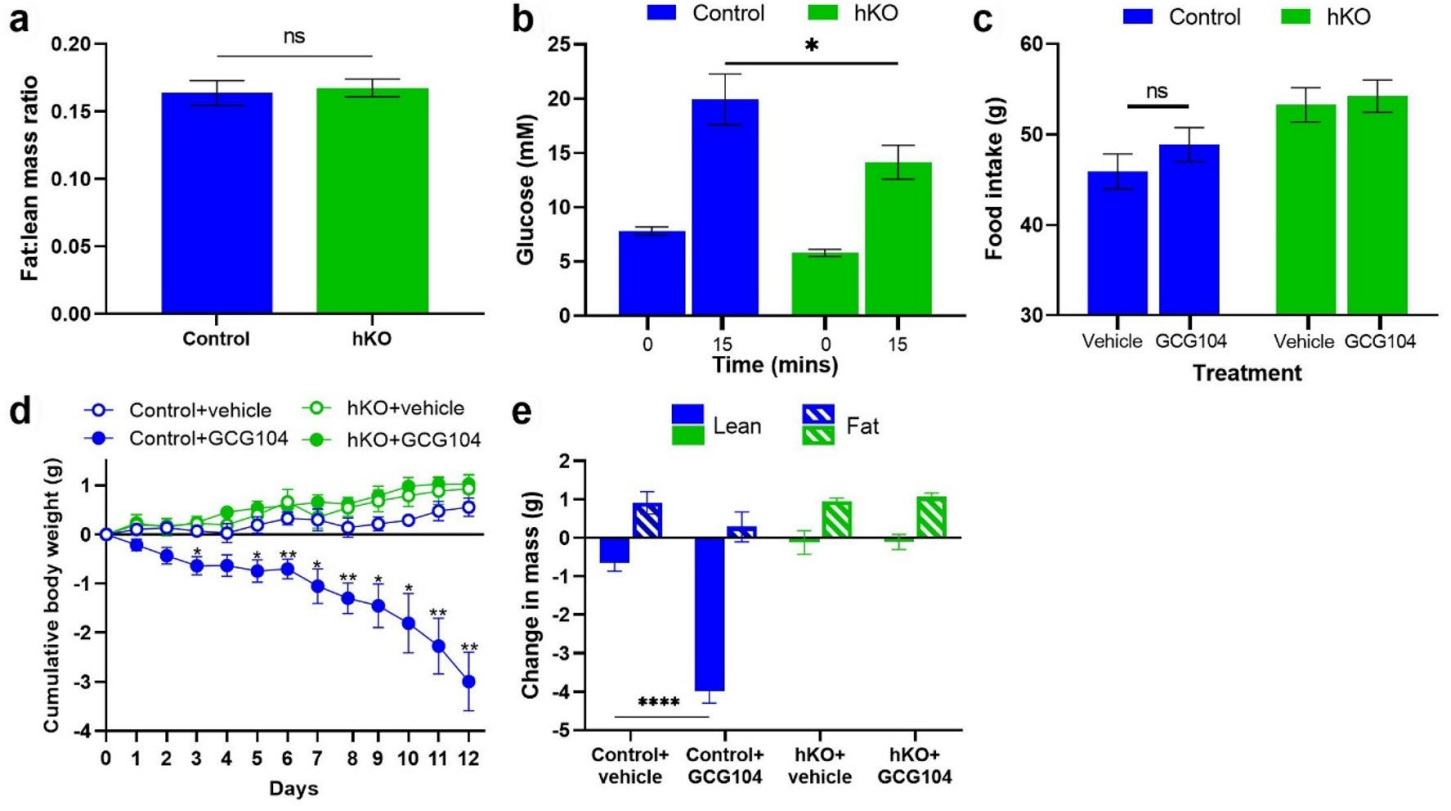
GCG104 reduces body weight in mice by hepatocyte signalling. (**a**) Fat:lean mass ratio of the hKO mice and controls (n = 13 per group; unpaired *t* test). (**b**) Baseline and 15-min glucose response readings for an IPGTT in the hKO (n = 8) and control mice (n = 5; two-way ANOVA with Sidak post hoc test). (**c**) 12-day cumulative food intake for control and hKO animals injected daily with vehicle (n = 4 and 7, respectively) or 10 nmol/kg GCG104 (n = 5 and 7, respectively; two-way ANOVA with Sidak post hoc test). (**d**) 12-day cumulative body weight change in control and hKO animals injected daily with vehicle (n = 4 and 7, respectively) or 10 nmol/kg GCG104 (n = 5 and 7, respectively; repeated measures 2-way ANOVA with Tukey’s post hoc test). (**e**) 12-day change in body composition for control and hKO animals dosed with vehicle (n = 4 and 7, respectively) or 10 nmol/kg GCG104 (n = 5 and 7, respectively); two-way ANOVA with Sidak post hoc test. *p* values indicated by *p < 0.05, **p < 0.01, ****p < 0.0001. All data presented as mean ± SEM.

The hepatic GCGR has previously been suggested as the mediator of chronic glucagon-induced weight loss^16^. To confirm whether the liver is the primary site of GCG104-induced weight loss, we first generated mice with deletion of the *Gcgr* specifically in liver hepatocytes (‘hKO’ mice). Unlike the cKO mice, the hKO animals developed with a normal fat:lean mass ratio compared to their control littermates (Fig. 3a). This is consistent with the suggestion that central GCGR signalling may be responsible for maintaining normal body composition^20^. hKO animals also had lower baseline blood glucose and improved glucose tolerance (Fig. 3b), as seen in cKO animals (Fig. 2b), and as previously reported^16,18^.

A pilot study surprisingly demonstrated that daily dosing with 7.5 nmol/kg GCG104 (which reduced body weight in the controls of the cKO mice (Fig. 2d)), had no effect on body weight in the controls of the hKO mice (Supplementary Fig. 4). We therefore increased the dose to 10 nmol/kg. This dose had no effect on food intake (Fig. 3c) but caused significant weight loss in control animals (Fig. 3d and Supplementary Fig. 2b) that was exclusively a result of decreased lean-mass (Fig. 3e and Supplementary Fig. 3b). Neither food intake, body weight, nor body composition were affected in hKO mice (Fig. 3c–e). This experiment confirms that hepatic GCGR action is responsible for weight loss at this dose of GCG104.

In this study, we have developed and tested a novel long-acting GCGR agonist to probe the tissue origin of weight-lowering effects resulting from chronic GCGR activation in mice. Our experiments have confirmed the liver as the target organ for glucagon-induced weight loss in this context. They also support the notion that signalling in the liver somehow increases energy expenditure, as GCG104-treated animals lost weight despite no change in food intake (Fig. 3). That said, it is not yet clear exactly how GCGR agonists increase energy expenditure; increased thermogenesis in the brown adipose tissue^23^, increasing locomotor activity^13^, or futile cycling of metabolites^2,24^, have all been suggested.

A molecular explanation for apparent differences in GCG104-sensitivity in the controls of our two cohorts of transgenic mice is currently lacking. However, although both cohorts were of the same C57BL/6 J background strain, it is possible that their variable GCG104-sensitivity may have been due to genetic drift. Previously it has been shown that various parameters of glucose homeostasis can indeed be influenced by mouse strain^25^, but the impact of subtle genetic differences like what may have occurred here has yet to be explored.

In any case, these studies have provided important insights into the biology of glucagon receptor agonism. We have shown that GCG104 differentially affects body composition in the ‘peri-anorectic’ range, possibly due to interindividual variation (Figs. 2, 3 and Supplementary Fig. 3). Indeed, our data point to at least one ‘tipping-point’ of glucagon action; that on food intake. However, there may be other specific doses that interact with individual sensitivity to trigger additional physiological events such as sympathetic activation. Finally, our data raise questions about the clinical utilisation of GCGR agonism: the fact that subtle differences in dose/sensitivity can alter its biology suggests that some level of personalization may be clinically advantageous. Indeed, this is supported by suggestions that diabetes and fatty liver disease can alter glucagon sensitivity in humans^26^.

## Supplementary Information

Supplementary Figures.
