# Supplementary material for: A glucagon analogue decreases body weight in mice via signalling in the liver
Source: Sci Rep. 2021 Nov 19;11:22577. doi: 10.1038/s41598-021-01912-0 (PMC8604983; doi:10.1038/s41598-021-01912-0)
Supplement: Supplementary file 1 — Supplementary Figures. [file 41598_2021_1912_MOESM1_ESM.docx]

**A Glucagon Analogue Decreases Body Weight in Mice Via Signalling in the Liver**

Charlotte E. Hinds^1+^, Bryn M. Owen^1+^, David C. D. Hope^1^, Philip Pickford^1^, Ben Jones^1^, Tricia M. Tan^1^, James S. Minnion^1^, Stephen R. Bloom^1^*

^1^Section of Investigative Medicine, Department of Metabolism, Digestion, and Reproduction, Imperial College London. W12 0NN

^+^Contributed Equally
*For correspondence s.bloom@imperial.ac.uk

**Supplementary Data**


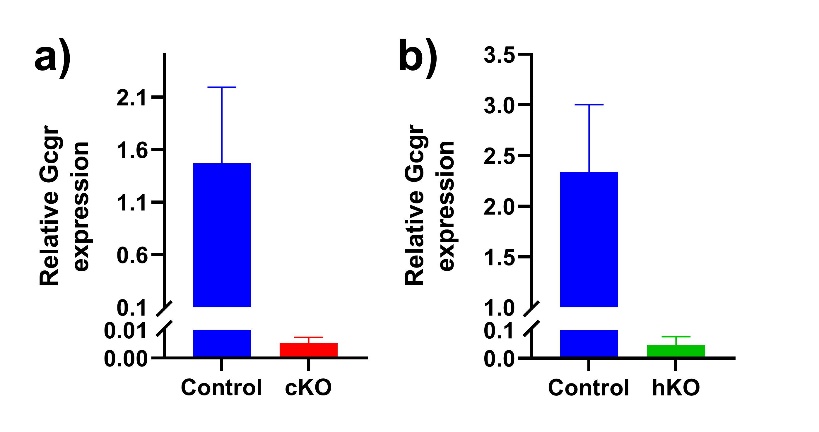


**Supplementary Figure 1 – confirmation of knockout in the cKO and hKO animals.** Relative *Gcgr* expression in the liver tissue of the control and knockout animals of a) the cKO cohort (n=3-5) and b) the hKO cohort (n=4) as determined by qPCR. All data presented as mean ± SEM.


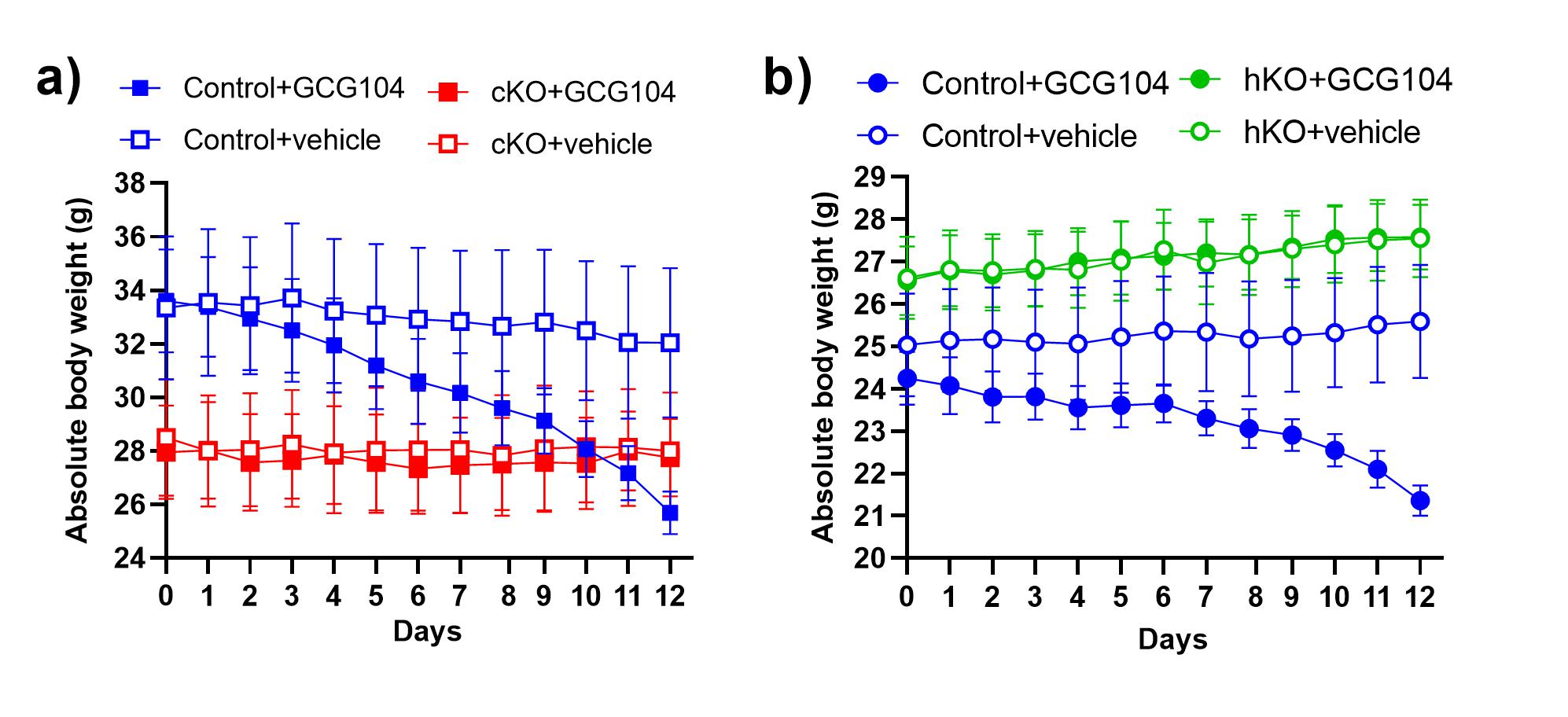


**Supplementary Figure 2 – GCG104 reduces absolute body weight in the control animals of both cohorts.** 12-day body weight change during daily administration of GCG104 at a) 7.5 nmol/kg in the cKO cohort (n=5-6) and b) 10 nmol/kg in the hKO cohort (n=4-7). Data presented as mean ± SEM.

**
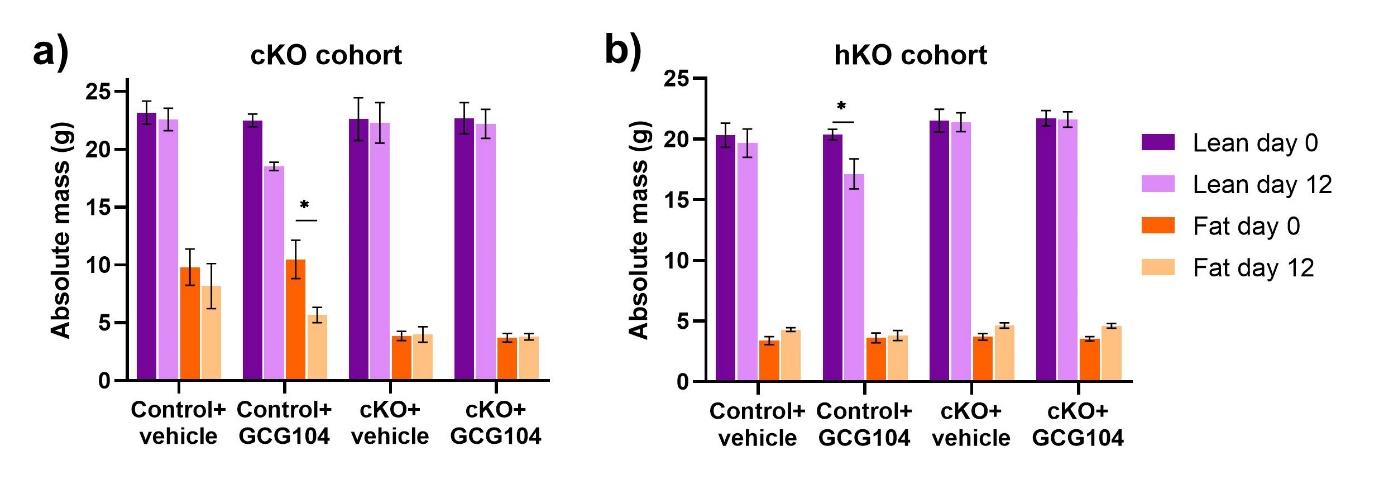
**

**Supplementary Figure 3 – Chronic GCG104 differentially affects body composition in the control animals of the cKO and hKO cohorts.** 12-day body composition changes following chronic administration of GCG104 at a) 7.5 nmol/kg in the cKO cohort (n=4-7) and b) 10 nmol/kg in the hKO cohort (n=5-7). *p<0.05. Data presented as mean ± SEM and analysed by two-way ANOVA with Tukey’s post hoc tests.


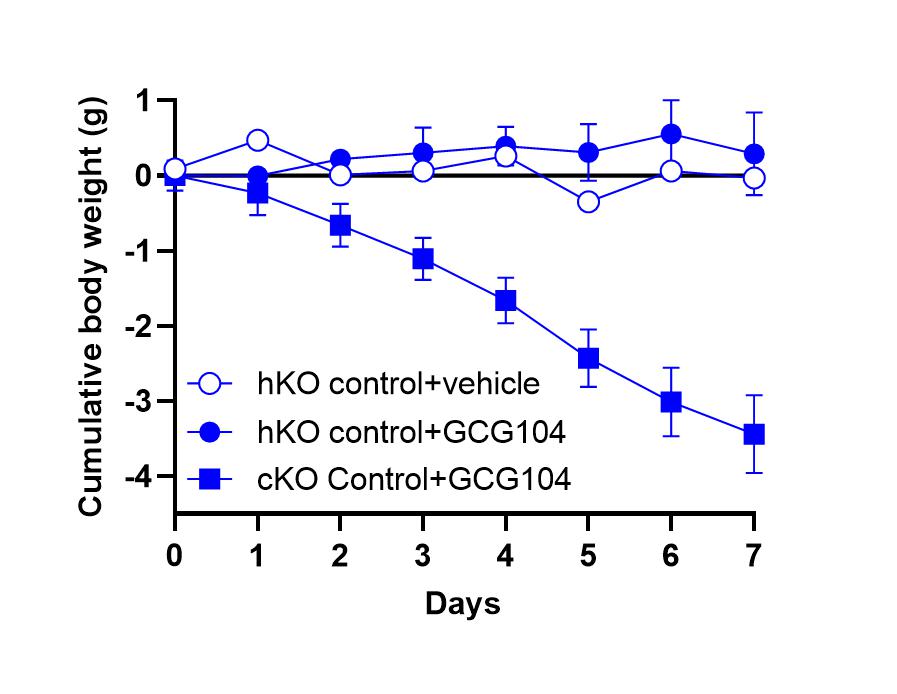

**Supplementary Figure 4 - GCG104 at a dose of 7.5 nmol/kg does not affect body weight in the hKO genetic background.** 7-day pilot study in hKO control littermates with daily vehicle (n=1) or 7.5 nmol/kg GCG104 (n=2) administration. Data from GCG104-dosed cKO control littermates is superimposed from Figure 2 for comparison (n=5). All data presented as mean ± SEM.
